# A Novel De Novo *STAG1* Variant in Monozygotic Twins with Neurodevelopmental Disorder: New Insights in Clinical Heterogeneity

**DOI:** 10.3390/genes15091184

**Published:** 2024-09-09

**Authors:** Lorenzo Cipriano, Roberta Russo, Immacolata Andolfo, Mariangela Manno, Raffaele Piscopo, Achille Iolascon, Carmelo Piscopo

**Affiliations:** 1 Department of Molecular Medicine and Medical Biotechnology, University Federico II, 80131 Naples, Italy; lorenzo.cipriano@unina.it (L.C.); roberta.russo@unina.it (R.R.); immacolata.andolfo@unina.it (I.A.); manno@ceinge.unina.it (M.M.); achille.iolascon@unina.it (A.I.); 2 CEINGE Biotecnologie Avanzate Franco Salvatore, 80145 Naples, Italy; 3 Department of Neuroscience, Reproductive and Odontostomatological Sciences, University Federico II, 80131 Naples, Italy; raffaele.piscopo2@unina.it; 4 Medical and Laboratory Genetics Unit, A.O.R.N. “Antonio Cardarelli”, 80131 Naples, Italy

**Keywords:** cohesin complex, cohesinopathies, STAG1, STAG2, SMC3, SMC1A, RAD21, Cornelia de Lange

## Abstract

Background: The *STAG1* gene encodes a component of the cohesin complex, involved in chromosome segregation and DNA repair. Variants in genes of the cohesin complex determine clinical conditions characterized by facial dysmorphisms, upper limb anomalies, intellectual disability, and other neurological deficits. However, to date, the *STAG1*-related clinical phenotype has been poorly investigated (around 20 cases reported). Methods and Results: We report, for the first time, two twins affected by a syndromic neurodevelopmental disorder associated with a de novo variant in the *STAG1* gene. Although both the twins showed a neurodevelopmental delay, one of them showed a more severe phenotype with greater behavioral problems, speech defects and limb apraxia. CGH array showed a 15q13.3 microduplication, inherited from an unaffected mother. Conclusions: We found different degrees of behavioral, speech and cognitive impairment in two twins affected by a neurodevelopmental disorder associated with a *STAG1* variant. These findings highlight the variability of the *STAG1*-associated phenotype or a probable role of associated variants (like the discovered 15q13.3 microduplication) in modulating the clinical features.

## 1. Introduction

The cohesin complex is an evolutionary-conserved structure involved in chromosome segregation during cell division. Although its primary role is to mediate sister chromatid cohesion and ensure proper chromosome segregation [1,2], recent studies have shown that this complex is also involved in other biological processes, such as DNA damage checkpoint and repair, DNA replication, gene transcription regulation, and chromatin structure maintenance [3].

The cohesin complex consists of three main subunits, SMC3, SMC1A and RAD21, along with a group of HEAT repeat-containing proteins, including STAG1 and STAG2 [3]. Additionally, several regulating proteins (NIPBL, ESCO2, HDAC8, DDX11, SGOL1, WAPL, PDS5A, PLK1, AURKB and ATRX) are responsible for mediating the interactions between this complex and the chromatin [3,4]. 

Variants in these genes, encoding for proteins part of the cohesin complex, have been associated with syndromic disorders that share some common clinical features, leading to the identification of a new group of clinical conditions described under the term “cohesinopathies”. Cornelia de Lange syndrome is the archetype of these conditions and has been associated with pathogenic variants in the three principal genes of the cohesin complex (i.e., *SMC3*, *SMC1A*, *RAD21*) [2,5,6]. Variants in the *STAG2* gene have been associated with Mullegama–Klein–Martinez syndrome, a condition characterized by the association of dysmorphic facial features, microcephaly, hearing loss, development delay, congenital heart defect and digit anomalies [5]. In addition, pathogenic variants in some of these genes of the cohesin complex (e.g., *SMC1A*, *STAG2*) have been associated with syndromic and non-syndromic neurological phenotypes, commonly related to structural anomalies at brain MRI, such as X-linked holoprosencephalies or epileptic encephalopathies with or without midline brain defects, highlighting the crucial role of the cohesin complex in brain development and function. 

Overall, all these conditions share some clinical features such as characteristic facial dysmorphisms, microcephaly, growth retardation, developmental delay, intellectual disability, upper limb anomalies, and some other neurological deficits such as epilepsy, autism, and non-specific brain MRI alterations [6].

Concerning the *STAG1* gene, only recently, a case series describing 17 patients with deletions or point variants in this gene shed some light on the main clinical features of the *STAG1*-related phenotype [7]. In the following years, two other reports of de novo pathogenic variants in the *STAG1* gene [8,9] contributed to the better definition of this condition’s clinical variability. 

Here, we report, for the first time, two monozygotic twins affected by a syndromic neurodevelopmental disorder associated with a de novo variant in the *STAG1* gene.

## 2. Case Description

**Figure 1:**
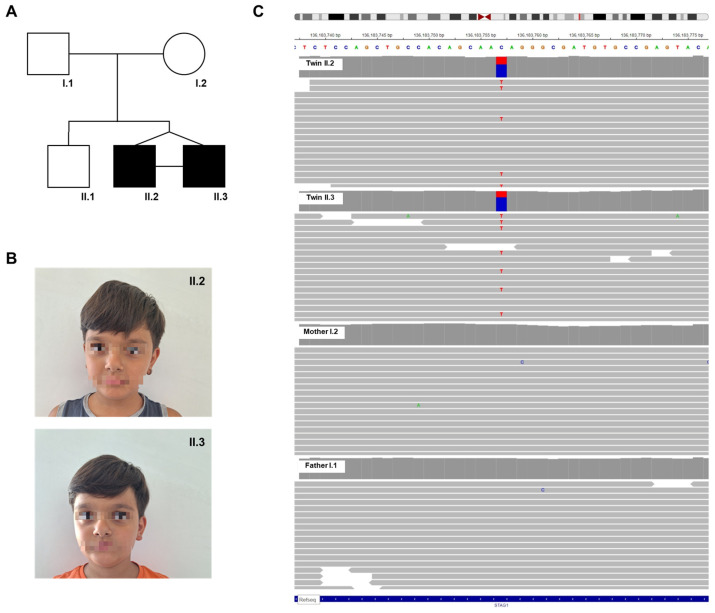
Phenotypic and genetic analysis. (**A**) Family pedigree: the diagram includes squares for male family members and circles for female members; individuals affected by the condition are represented by solid symbols. (**B**) Facial appearance of patient II.2 (**upper** panel) and patient II.3 (**bottom** panel). (**C**) Genomic visualization: alignment of the genomic region containing the *STAG1* variant for all family members, analyzed using the seqr software (seqr v1.0-956f348a).

Two 9-year-old monozygotic twins were admitted to our department for evaluation of a syndromic neurodevelopmental disorder. Their family history was negative for neurodevelopmental disorders; their older brother was a healthy 22-year-old man and neither parent reported developmental delays during their childhood (Figure 1A).

The pregnancy was unremarkable and prenatal screenings, including biochemical and ultrasound investigations, were normal. At birth, due to their prematurity (week 33), they were admitted to the neonatal intensive care unit where they underwent extensive metabolic screening, neonatal cranial sonography, ophthalmologic evaluation, and auditory evoked potentials, all of which were normal. Both children stood up at normal age and began walking at 11 months. Control of the sphincter was acquired at the age of 3 for both twins. Concerning language development, twin II.3 began speaking at 48 months, while twin II.2 started at 40 months. At the time of clinical evaluation, both children had a diagnosis of intellectual disability, with a more severe impairment in twin II.3, significantly affecting daily life activities. Both had an extensive vocabulary, but twin II.3 exhibited pronounced speech difficulties, mainly characterized by agrammatism and phonetic errors. Mild differences were also evident in terms of behavior. Indeed, twin II.3 developed marked behavioral problems, mainly progressive social isolation, emotional distress, hyperactivity, and frequent episodes of public soliloquy. In contrast, twin II.2 did not present relevant behavioral problems and demonstrated excellent interaction with peers. Overall, they are currently (at the age of 9) able to perform basic daily activities and independently use modern technological devices. They can make calls, send messages, play video games and properly use computers.

At clinical evaluation, both the probands shared some clinical features such as deep-set eyes, hypertelorism, thin eyebrows, a high nasal bridge, a bulbous tip, a deep philtrum, a pronounced Cupid’s bow, microdontia with mild gingival hyperplasia, bilateral flat foot, hyperlaxity and obesity (HP:0000490, HP:0000316, HP:0045074, HP:0000426, HP:0000414, HP:0002002, HP:0006311, HP:0000212, HP:0001763, HP:0001382) (Figure 1B). No differences, with the only exception of an everted lower lip vermilion in patient II.2, in facial morphology were found. Abdominal and cardiac ultrasounds, ophthalmological and auditory evaluations as well as brain imaging and EEG investigations did not show abnormalities. Endocrinological, complete blood count, and basic coagulation studies were also performed without finding pathologies or differences between the twins.

In the suspicion of a genetic etiology of the current disorder, both the patients underwent several genetic tests, i.e., karyotype, *FMR1* repeat expansion study and array-CGH. Karyotype and *FMR1* evaluations showed no abnormalities, while array-CGH identified a maternally inherited 417.2 kb duplication on chromosome 15 (chr15) that spans three exons of the *CHRNA7* gene. Although this gene is not linked to any known disease and the duplication has been noted in several asymptomatic individuals, it was deemed non-pathogenic. Consequently, a more comprehensive genetic assessment, including clinical exome analysis, was conducted for both probands and their parents. DNA samples were obtained from each subject after signed informed consent was acquired and according to the Declaration of Helsinki. Genomic DNA was extracted from peripheral blood using the Maxwell RSC Blood DNA Kit for automated extraction (Promega, Milan, Italy). To evaluate the quality of the extracted genomic DNA before fragmentation, samples were quantified using a UV-Vis spectrophotometer (NanoDrop 2000; Thermo Scientific, Waltham, MA, USA). Clinical exome (CES) analysis was conducted using a commercially available panel of over 5000 genes associated with hereditary diseases (SureSelect custom Constitutional Panel 17 Mb, Agilent Technologies, Santa Clara, CA, USA), as previously described [10,11]. Variant pathogenicity was assessed following the ACMG/AMP guidelines using a scaled point system [12,13]. Scores ranged from 0 to 5 for variants of unknown significance (VUS) and between 6 and 9 for likely pathogenic (LP) variants. Variants scoring 10 or higher were classified as pathogenic (P) [13]. 

Clinical exome analysis revealed a heterozygous variant NM_005862.3:c.1279G>A in the *STAG1* gene in both monozygotic twins, introducing a missense change p.Val427Ile (V427I) (rs1231019385, alternative allele frequency G = 0.000004, GnomAD_exome v2.1.1). The variant exhibited a slightly skewed alternative allele frequency in both twins (twin II.2: read depth 86, allelic balance 0.37; twin II.3: read depth 106, allelic balance 0.29). Segregation analysis showed the variant was not inherited from either parent (father’s read depth 92, mother’s read depth 122) (Figure 1C), indicating a de novo occurrence likely due to germline mosaicism in one of the parents or early postzygotic variation. Unfortunately, the older brother was unavailable for segregation analysis of the identified variant. According to the ACMG guidelines, the variant was classified as likely pathogenic.

In consideration of the recently reported possible role of *STAG1* alterations in predisposition to childhood hematological malignancies, we programmed a tailored hematological follow-up for our probands [14].

## 3. Discussion 

We herein describe for the first time a pair of twins affected by a neurodevelopmental disorder associated with a de novo variant in the *STAG1* gene. The two patients showed a different degree of intellectual disability, motor and speech delay and behavioral abnormalities, suggesting the clinical variability of the *STAG1*-associated phenotype. In detail, patient II.3 presented a more severe phenotype overall, with more pronounced articulatory and phonetic problems, limb apraxia, social isolation, and cognitive impairment compared to his brother.

To date, the exact function of the *STAG1* gene is poorly understood. As a subunit of the cohesin complex, *STAG1* appears to be essential for normal embryonic development, as evidenced by the finding that *STAG1* knockout mice display developmental defects and embryonic lethality [15]. The *STAG1*-related phenotype has been recently described by Lehalle et al. [7] who provided an accurate description of a series of 17 patients with syndromic intellectual disability associated with a variant in this gene. In 7 of the 17 cases, *STAG1* involvement was due to a CNV (always a deletion), which, in most cases, affected not only the *STAG1* but both this gene and the *PCCB* gene. In the CNV group, not all variants were de novo, and milder intellectual disability was observed in cases where the CNV was inherited from a parent. Cases with point variants showed a more variable degree of intellectual disability ranging from a very mild neurodevelopmental delay to a more severe cognitive impairment. Associated neurologic features were frequently described, encompassing seizures, autism spectrum disorder, behavioral anomalies and non-specific brain MRI findings (e.g., dilation of ventricles, global brain atrophy, Chiari malformation, etc.). The description of the case by Di Muro et al. [8] aligns with these results, showing a syndromic developmental delay with EEG anomalies. Therefore, the crucial aspect of the *STAG1*-related phenotype is represented by the intellectual disability/neurodevelopmental delay that is found in all the cases described so far. 

Although it is currently challenging to define a specific pattern of STAG1-related dysmorphic features (due to the limited number of cases and the significant variability in described facial traits), some degree of somatic phenotypic variation is always present. Common features include deep-set eyes, a wide mouth, a high nasal bridge, thin eyebrows and widely spaced teeth, several of which were also present in our patients (Table 1). However, due to the low specificity of these features and the few descriptions in the literature to date, a characteristic *STAG1* phenotype is still lacking. Feeding difficulties, seizures, autism spectrum disorders and both prenatal and/or postnatal growth retardation are commonly associated. Less frequent is systemic involvement, with cryptorchidism, scoliosis and congenital heart defects the most commonly associated anomalies. However, although both our patients manifested a neurodevelopmental disorder with some facial dysmorphisms, they did not show any of these structural anomalies. Although *STAG1*-associated disorders are part of the cohesinopathies, they do not share several features of Cornelia de Lange syndrome. The patients did not exhibit limb abnormalities, hypertrichosis, growth failure or other common dysmorphic traits such as synophrys, long and thick eyelashes, a long philtrum, a highly arched or cleft palate, micrognathia or a short nasal bridge. 

Another aspect that needs to be taken into account is the recently described malignancy predisposition. Saitta et al. have suggested a potential role of *STAG1* variants in genetic predisposition to childhood hematological malignancies [14]. This aspect implies that patients with *STAG1* pathogenic variants could benefit from long-term monitoring to promptly detect hematological malignancies. For this reason, we adopted a specific hematological follow-up for our probands. We suggested continuing the neuropsychiatric follow-up and the logopedic and psychomotor treatment, especially for patient II.3. We also recommended a new neurological evaluation with (if deemed appropriate by the specialist) a novel EEG and MRI study.

Considering the small duplication involving part of the *CHRNA7* gene, the CNV was considered non-causative of the disorder. Microduplications involving the *CHRNA7* gene have been associated with neurodevelopmental disorders, intellectual disability, epilepsy, autism spectrum disorder and attention deficit hyperactivity disorder as well as with other psychiatric symptoms (e.g., schizophrenia, Tourette syndrome, obsessive compulsive disorder) [16,17,18,19]. However, 15q13.3 microduplications are equally prevalent in patients and healthy controls [20,21,22], making it extremely difficult to classify this CNV as pathogenic. Additionally, microduplications as opposed to microdeletions tend to have a higher degree of variability in the expressivity and penetrance of these symptoms, making their clinical interpretation more difficult. In our cases, the duplication was inherited from a mother clinically unaffected with no family history of neurological or psychiatric diseases. However, the incomplete penetrance of the disorder or a variable expression in our probands cannot be excluded. This factor could potentially explain the mild phenotypic differences between the twins. However, the absence of functional investigations confirming or refuting the hypothetical interaction between the CNV and *STAG1* products is a limitation of this study, and any influence on the clinical phenotype remains speculative.

The clinical heterogeneity of the probands could also be associated with the presence of different somatic mosaicism in the twins. In fact, due to the unavailability of the older brother for segregation analysis and the absence of additional tissue samples of patients for testing, it is not possible to completely exclude this possibility.

In conclusion, we herein describe phenotypic differences in a pair of twins with a de novo variant in the *STAG1* gene. To our knowledge, this is the first description of a pair of twins affected by a *STAG1*-related disorder. The highlighted differences could be due to a different environment, an eventual mosaicism in the patients, or gene modifiers. Therefore, future descriptions, as well as molecular studies on the effect of possible gene modifiers, will be necessary to better understand the clinical variability of the *STAG1*-related conditions.

## Figures and Tables

**Table 1:** Clinical features of the probands.

Clinical Features	Patient II.3	Patient II.2
*STAG1*-related features according to [7]		
Epilepsy	−	−
EEG anomalies	−	−
Intellectual disability	mild–moderate	mild
Autistic features	+	−
Hyperlaxity	+	+
Brain imaging anomalies	−	−
Other neurological and psychiatric differences		
Behavioral disturbances	+	−
Speech delay (first word)	48 months	40 months
Speech problems (e.g., articulatory, agrammatism)	+	−
Limb apraxia	moderate	mild
Sphincter control	36 months	32 months
Hypotonia	−	−
Facial dysmorphisms previously described in the STAG1-associated conditions [7]		
High nasal bridge	+	+
Deep-set eyes	+	+
Wide mouth	−	+
Widely spaced central incisor	−	−
Thin eyebrows	+	+
Flat foot	+	+
Other features not included in the previous reports		
Small teeth	+	+
Gingival hyperplasia	+	+
Other investigations		
Abdomen ultrasound	−	−
Cardiac ultrasound	−	−
Endocrinological disorders	−	−
Coagulation defects	−	−
Systemic involvement	−	−

## Data Availability

The data that support the findings of this study are available from the corresponding author, C.P., upon reasonable request.
